# Interpretable machine learning model for predicting 5-Year postoperative recurrence risk in patients with stage III colon cancer using preoperative laboratory tests: a two-centre study

**DOI:** 10.1186/s12876-025-04511-9

**Published:** 2026-01-29

**Authors:** Hangping Wei, Xihao Fu, Yuanyuan Cheng, Li Xu, Xinkai Wu, ZhenXin Wang

**Affiliations:** 1 Department of Medical Oncology, The First Affiliated Hospital of Soochow University, 899 Pinghai Road, Suzhou, Jiangsu 215006 P.R. China; 2 Department of Medical Oncology, Dongyang Hospital Affiliated to Wenzhou Medical University, Dongyang, Zhejiang 322100 P.R. China; 3 Department of Colorectal Medicine, Cancer Hospital of the University of Chinese Academy of Sciences (Zhejiang Cancer Hospital), Hangzhou, Zhejiang 310000 P.R. China

**Keywords:** Laboratory test, Cancer recurrence, Colon cancer, Stage III, Machine learning model

## Abstract

**Background:**

Colorectal cancer (CRC) is one of the most prevalent malignant diseases worldwide and displays significant heterogeneity. The aim of this study was to investigate the application of machine learning algorithms to incorporate preoperative laboratory tests for predicting the 5-year recurrence risk in patients with stage III colon cancer (CC) postsurgery.

**Methods:**

This study included two patient cohorts: the Zhejiang Cancer Hospital CC cohort (ZCC set, *n* = 290), which served as the training cohort, and the Dongyang CC cohort (DYC set, *n* = 125), which was utilized as an external testing cohort. Univariate analysis was initially performed on the 48 preoperative laboratory tests and 15 clinical and pathological features within the training cohort to pinpoint potential predictors. Features with a p value less than 0.05 were incorporated, and six machine learning models—logistic regression, random forest, XGBoost, support vector machine (SVM), back propagation neural network (BP NET), and K-nearest neighbour (KNN)—were employed to develop a model for predicting the 5-year recurrence risk in patients with stage III colon cancer. The prediction efficacy was assessed by calculating the area under the curve (AUC) of the machine learning model using the external test dataset, and comparisons were performed via the DeLong test. Ultimately, the Shapley additive explanations (SHAP) algorithm was applied to rank feature importance and compute the SHAP values for each feature, which were then visualized.

**Results:**

Univariate analysis identified 10 laboratory tests and 6 clinical and pathological features that were incorporated into six machine learning models. The random forest model exhibited the highest predictive performance in the test cohort, with an AUC of 0.845. Logistic regression closely trailed, achieving an AUC of 0.823. The DeLong test revealed that the predictive performance of the random forest model was comparable to that of logistic regression and outperformed the other models. SHAP analysis indicated that the most important feature for predicting the 5-year recurrence risk of stage III colon cancer was perineural invasion, followed by FIB and then PT.

**Conclusions:**

A machine learning model constructed using preoperative laboratory tests and clinical and pathological features can assist in predicting the 5-year recurrence risk of patients with stage III colon cancer. This model provides potential reference values for the clinical development of individualized treatment strategies.

**Supplementary Information:**

The online version contains supplementary material available at 10.1186/s12876-025-04511-9.

## Introduction

Colorectal cancer (CRC) represents a formidable global health challenge; it is the third and second most prevalent cancer in men and women, respectively [1–3]. It accounts for approximately 10% of all newly diagnosed malignancies and contributes to 9.4% of all cancer-related deaths worldwide, posing a significant public health burden [1, 4]. In recent years, propelled by rapid advancements in basic and clinical research, widespread implementation of early screening techniques, continuous refinement of comprehensive treatment concepts, and remarkable improvements in surgical techniques, significant progress has been made in the diagnosis and treatment of colon cancer (CC), leading to a substantial increase in patient survival rates [5, 6]. Nevertheless, postoperative recurrence remains the primary challenge in the current treatment of CC.

Among its subtypes, stage III CC has a particularly poor prognosis, with a 5-year recurrence rate of approximately 30% [7–9]. Surgical resection remains the cornerstone of curative treatment for stage III CC, with adjuvant chemotherapy being the standard of care due to the increased risk of disease recurrence [7, 10]. Unfortunately, even after undergoing standard treatment, some patients with stage III CC face unfavourable outcomes, and recurrence may occur even during the adjuvant treatment process [11].

The clinical assessment of postoperative recurrence risk in patients with CC predominantly currently relies on the TNM staging system. Although this system provides crucial prognostic insights, it has notable limitations in accurately predicting recurrence. Moreover, the stage III CC patient population exhibits significant heterogeneity, even within the same TNM stage [12, 13]. This gap in risk assessment highlights the urgent need for more sophisticated, multifaceted approaches that transcend traditional staging to better inform adjuvant management strategies and improve patient outcomes [14]. For high-risk patients, a more aggressive chemotherapy regimen can be adopted, or the postoperative monitoring strategy can be strengthened.

Against this backdrop, clinical prediction models have become a research hotspot. These models are mathematical constructs—parametric, semiparametric, or nonparametric—that forecast the unknown using known features. They function as quantitative tools to evaluate risks and benefits in medical decision-making, and their use is becoming increasingly widespread. In contrast to traditional learning, machine learning can identify complex nonlinear relationships within a broad spectrum of medical datasets and can adapt continuously to new data, thereby increasing the accuracy of prediction models [15–18]. Previous research has indicated that certain preoperative laboratory indicators and clinical and pathological features are associated with the recurrence of CC [19, 20]. However, previous studies have analysed only a few specific indicators, omitting the outcomes of all routine examinations, and have primarily used traditional regression analysis. There has also been no further analysis of the models’ interpretability.

The present study aimed to predict the 5-year recurrence risk following CC surgery by combining 48 preoperative routine laboratory test results with 15 clinical and pathological features. Furthermore, it compared and evaluated the predictive performance of six machine learning models and employed Shapley additive explanations (SHAPs) for model interpretation.

## Materials and methods

### Patients

This study included cohorts from two centres. The first cohort consisted of patients with pathological stage III CC who underwent curative surgical resection at Zhejiang Cancer Hospital between January 2015 and December 2019 and served as the training cohort (ZCC set, *n* = 290). The second cohort consisted of pathological stage III CC patients who underwent surgical resection at Dongyang Hospital affiliated with Wenzhou Medical University between January 2013 and December 2019 and was used as the external testing cohort (DYC set, *n* = 125). All enrolled patients signed an informed consent form prior to surgery and underwent standard surgical resection. Complete pathological reports confirmed stage III disease (i.e., positive lymph nodes). Other inclusion criteria also included an age range of 18 to 90 years, the absence of coexisting of malignant tumours in other parts of the body, missing data not exceeding 30%, the absence of antitumour treatment before surgery, and complete follow-up data.

### Data collection

Clinical and pathological features were extracted from electronic medical records and standardized pathology reports. These factors included age, gender, body mass index (BMI), tumour site, pT stage, pN stage, overall stage, maximum diameter, histological grade, histological type, number of lymph nodes dissected, vascular invasion, perineural invasion, adjuvant chemotherapy regimen, and number of chemotherapy cycles. The adjuvant treatment regimens were classified into three categories: no adjuvant treatment, monotherapy, and combination therapy. The number of treatment cycles was grouped into three categories: 0 cycles, fewer than 4 cycles, and 4 or more cycles.

The missing data for D-dimer, which accounted for 22.1%, was excluded from the analysis, resulting in 48 laboratory tests. These tests encompassed six categories: routine blood examination, liver and kidney function tests, lipid metabolism, tumour markers, coagulation function, and other derived parameters. The other derived parameters include the prognostic nutritional index (PNI), systemic immunoinflammatory index (SII), platelet-to-lymphocyte ratio (PLR), and neutrophil-to-lymphocyte ratio (NLR), which are four metrics in total. The specific calculation formulas for these parameters are as follows: PNI = Alb (g/L) + 5 × absolute lymphocyte count (10^9/L), SII = absolute platelet count (10^9/L) × absolute neutrophil count (10^9/L)/absolute lymphocyte count (10^9/L), NLR = absolute neutrophil count (10^9/L)/absolute lymphocyte count (10^9/L), and PLR = absolute platelet count (10^9/L)/absolute lymphocyte count (10^9/L). All indicators represent the last results obtained within one week before surgery.

### Follow-up

According to the NCCN guidelines, patients with stage III colon cancer are recommended to undergo six months of oxaliplatin-based adjuvant chemotherapy, such as mFOLFOX6 (5-fluorouracil, leucovorin, and oxaliplatin) or XELOX (capecitabine and oxaliplatin) [7]. However, the decision to undergo chemotherapy, along with the choice of regimen and cycle, was made collaboratively between the patient and the physician, considering the patient’s baseline condition, tolerance, and preferences. We provided outpatient follow-up every 3‒6 months after surgery. The last follow-up period for all patients was January 31, 2025, with a minimum follow-up duration of over 5 years. The definition of a recurrence event refers to the confirmation by comprehensive clinical judgment or histopathological examination of definite recurrent signs detected through clinical assessment methods such as imaging examinations and tumor marker tests in patients after radical resection.

### Univariate and machine learning analysis

**Fig. 1:**
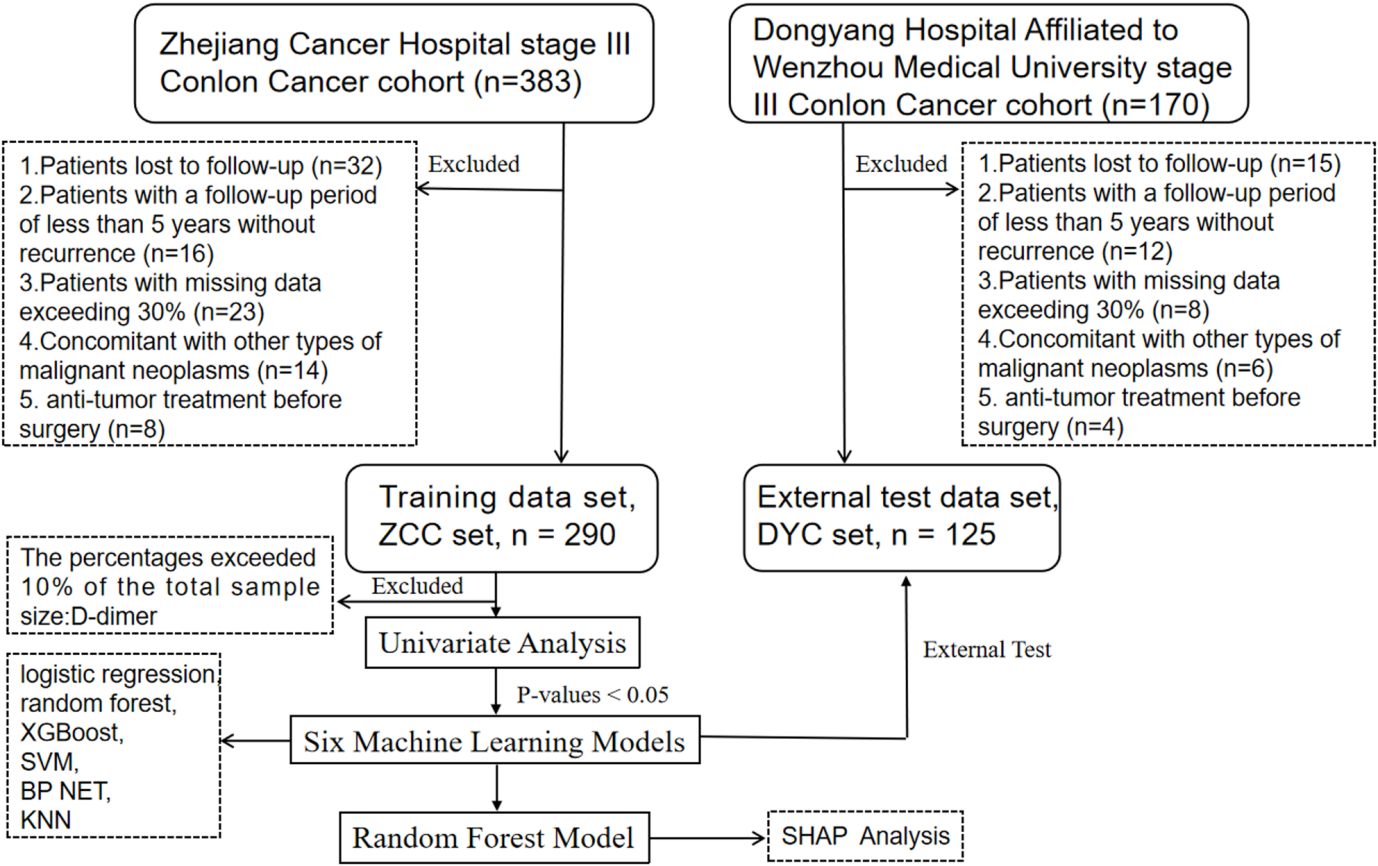
Flowchart of data collection and filtering in two cohorts

Features with missing data exceeding 10% of the dataset were excluded (with the D-dimer parameter being the only one removed), and the remaining features with missing data were addressed using multiple imputation. Multiple imputation was performed using the mice package in R, which enables hybrid imputation for handling multiple types of variables. Specifically, the following imputation methods were applied based on variable types: (a) predictive mean matching (pmm) was used for numerical data; (b) logistic regression (logreg) was employed for binary factor variables; (c) multinomial logistic regression (polyreg) was utilized for unordered multi-level factor variables; and (d) the proportional odds model (polr) was adopted for ordered multi-level factor variables. Univariate analysis was performed on all the features in the training cohort to identify potential predictors. Features with a p value less than 0.05 were incorporated, and six machine learning models—logistic regression, random forest, XGBoost, support vector machine (SVM), back propagation neural network (BP NET), and K-nearest neighbour (KNN)—were employed to predict 5-year recurrence. For machine learning processing, 5-fold cross-validation was applied. The caret R package (R version 4.2.0) was used to build all machine learning models, incorporating the “stat” R package for logistic regression, the “randomForest” R package for random forest, the “xgboost” R package for XGBoost, the “e1071” R package for SVM, the “nnet” R package for BP NET and the “KKNN” R package for KNN. To evaluate each model’s performance, we used metrics such as the area under the curve (AUC), accuracy, sensitivity, and specificity. The DeLong test was conducted to compare their predictive powers. To enhance the interpretability of the machine learning models, the optimal model was subsequently selected from the six aforementioned machine learning models. The SHAP method was applied for interpretation, including ranking feature importance, calculating SHAP values for each feature, and visualizing these values to clarify the specific associations between features and the 5-year recurrence risk of patients. The SHAP package (Python version 3.12.8) was used. A flowchart outlining the cohorts used in this study is shown in Fig. 1.

### Statistical analysis

All analyses were conducted using R statistical software (version 4.2.0) and Python statistical software (version 3.12.8) for Windows. The Shapiro–Wilk test was applied to assess the normality of the distribution of clinical features within the cohorts. Continuous data are expressed as the means ± standard deviations or medians (interquartile ranges), with differences analysed using t tests or Mann‒Whitney U tests, as appropriate. Categorical data are presented as frequencies or percentages, and differences were analysed using the chi-square test or Fisher’s exact test. A p value of < 0.05 was considered to indicate statistical significance.

## Results

### Baseline clinical and pathological conditions

Patients lost to follow-up, those with a follow-up period of less than 5 years without recurrence, and patients with missing data exceeding 30% were excluded. A total of 415 patients from two cohorts were included in this study. Of these, 290 patients from Zhejiang Cancer Hospital formed the training set (ZCC set), and 125 patients from Dongyang Hospital affiliated with Wenzhou Medical University constituted the external testing set (DYC set). In the training set the number of patients with and without recurrence was 104 and 186, respectively. In the external testing set, the number of patients with and without recurrence was 63 and 62, respectively. The clinical and pathological features of the two cohorts were largely consistent, as detailed in Table 1. Compared with the training cohort, the test cohort clearly had a greater proportion of elderly patients, larger tumour diameters, later tumour stages, fewer lymph node dissections, more absences of adjuvant chemotherapy, and fewer chemotherapy cycles. These factors may have contributed to the higher recurrence rate in the test cohort. The results of multiple imputation for missing data in the two cohorts are presented in the supplementary materials (Table S1).

**Table 1:** Baseline clinical and pathological features of patients in the two cohorts

Features	Total (n=415)	Training cohort (n=290)	Test cohort (n=125)	**p value**
age	61(53.5,70)	59(51,67)	65(55,74)	p<0.05
gender				p=0.05
male	222(53.5)	166(57.2)	56(44.8)	
female	193(46.5)	124(42.8)	69(55.2)	
BMI				p=0.10
≤18.4	20(4.8)	11(3.8)	9(7.2)	
18.5–23.9.5.9	251(60.5)	169(58.3)	82(65.6)	
≥24	144(34.7)	110(37.9)	34(27.2)	
tumour site				p=0.96
right	264(63.6)	186(64.1)	78(62.4)	
left	151(36.4)	104(35.9)	47(37.6)	
maximal diameter				p=0.99
≤5cm	264(63.6)	189(65.2)	75(60)	
>5cm	148(35.7)	99(34.1)	49(39.2)	
NA	3(0.7)	2(0.7)	1(0.8)	
histological type				p=0.96
adenocarcinoma	322(77.6)	221(76.2)	101(80.8)	
special type adenocarcinoma	93(22.4)	69(23.8)	24(19.2)	
histological grade				p=0.90
G1	5(1.2)	4(1.4)	1(0.8)	
G2	328(79.0)	222(76.5)	106(84.8)	
G3	82(19.8)	64(22.1)	18(14.4)	
pT stage				p=0.94
T1-2	16(3.9)	13(4.5)	3(2.4)	
T3	128(30.8)	96(33.1)	32(25.6)	
T4	271(65.3)	181(62.4)	90(72.0)	
pN stage				p=0.96
N1	291(70.1)	204(70.3)	87(69.6)	
N2	124(29.9)	86(29.7)	38(30.4)	
overall stage				p=0.84
IIIA	12(2.9)	10(3.4)	2(1.6)	
IIIB	300(72.3)	209(72.1)	91(72.8)	
IIIC	103(24.8)	71(24.5)	32(25.6)	
vascular invasion				p=0.91
0	247(59.5)	182(62.7)	65(52.0)	
1	156(37.6)	108(37.3)	48(38.4)	
NA	12(2.9)	0(0)	12(9.6)	

### Univariate analysis in the training set (ZCC set)

The missing data for D-dimer accounted for 22.1% of the dataset (64 out of 290), and since this exceeded 10% of the total sample size, they were excluded from the analysis. As a result, 48 laboratory tests and 15 clinical and pathological features, including postoperative adjuvant treatment status, were retrieved and subjected to univariate analysis. The P values for 16 features (10 laboratory tests and 6 clinical and pathological features) were less than 0.05, as detailed in Table 2. The 10 laboratory tests included NEU (neutrophil count), WBC (white blood cell count), RDW (red blood cell distribution width), ALP (alkaline phosphatase), TT (thrombin time), FIB (fibrinogen), PT (prothrombin time), CEA (carcinoembryonic antigen), NLR (neutrophil-to-lymphocyte ratio), and the SII (systemic immune-inflammation index). The 6 clinical and pathological features included age, pN stage, overall stage, perineural invasion, adjuvant chemotherapy regimen, and number of chemotherapy cycles. The P values of the remaining features were ≥ 0.05 and are presented in the supplementary materials (Table S2).

**Table 2:** Metrics for predicting 5-year recurrence risk in training and testing cohorts using six machine learning models

**Features**	**OR** **(** **95%CI** **)**	**p value**
age	1.0317(1.0225-1.0409)	0.0046
pN stage	1.9022(1.8480-1.9579)	0.0147
overall stage	2.6637(2.6627-2.6646)	<0.001
perineural invasion	4.3492(4.3492-4.3492)	<0.001
adjuvant chemotreatment regimen	0.5463(0.5460-0.5467)	<0.001
number of chemotreatment cycles	0.5920(0.5899-0.5940)	0.0018
NEU	1.2711(1.2678-1.2743)	0.0013
WBC	1.1859(1.1662-1.2059)	0.0085
RDW	0.9021(0.8663-0.9394)	0.0207
ALP	1.0176(1.0093-1.0258)	0.0041
TT	0.8137(0.7926-0.8355)	0.0134
FIB	1.5461(1.5345-1.5577)	0.0038
PT	1.3815(1.3699-1.3931)	0.0043
CEA	1.0085(0.9250-1.0996)	0.0441
NLR	1.1997(1.1871-1.2125)	0.0054
SII	1.0005(0.9933-1.0078)	0.0037

### Construction and evaluation of machine learning models

**Fig. 2:**
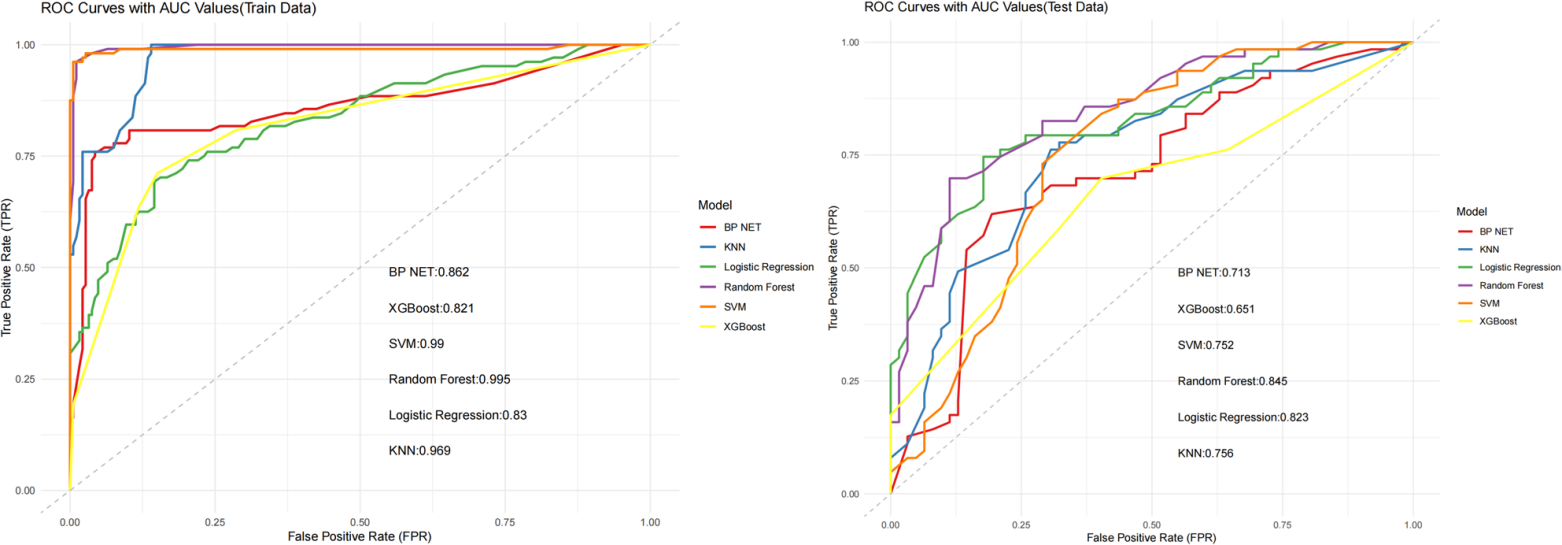
ROC curves of six different machine learning models in training and testing cohorts

**Fig. 3:**
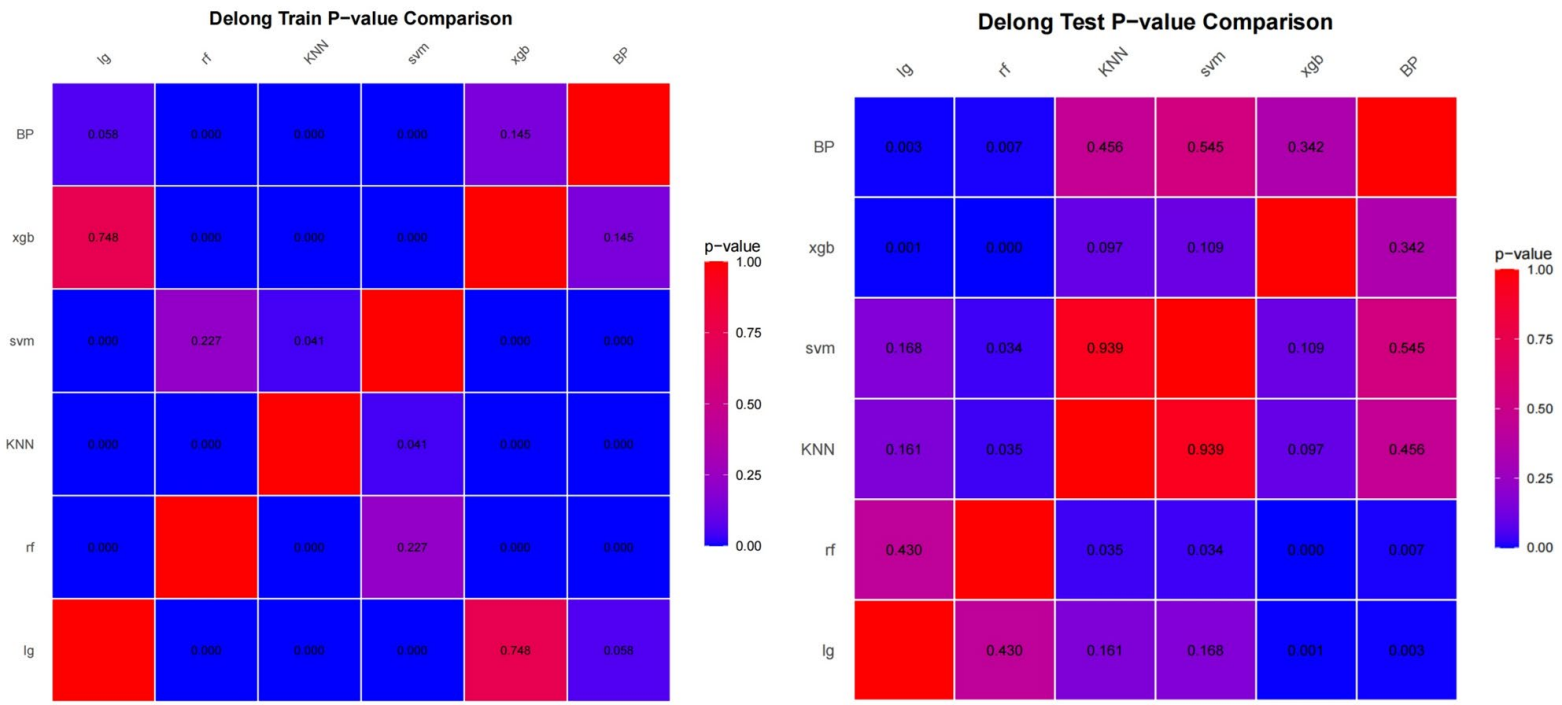
Heatmap of the delong test in training and testing cohorts (LR: logistic regression, RF: random forest)

The 16 features that exhibited statistical significance in the univariate analysis were utilized as inputs for six distinct machine learning models—specifically, logistic regression, random forest, XGBoost, SVM, BP NET, and KNN—to facilitate learning within the training cohort. The specific parameters of the six machine learning models are provided in supplementary materials A. The ability of these six machine learning models to predict the 5-year recurrence risk of stage III colon cancer was subsequently assessed in the external testing cohort (DYC set). The results indicated that the random forest model attained the highest AUC value, 0.845, followed by the logistic regression model, with an AUC of 0.823. Both models exhibited robust predictive capabilities. The results of the random forest with 5-fold cross-validation are presented in the supplementary materials (Table S3). The error rate ranged from 0.25 to 0.30, and the accuracy ranged from 0.70 to 0.75, indicating good model stability. In comparison, the predictive performance of the BP NET, SVM, and KNN models was moderate, with AUC values ranging from 0.713 to 0.756, whereas XGBoost had poorer predictive performance, with an AUC of 0.651, as illustrated in Fig. 2. The metrics for the training and testing cohorts for the prediction of 5-year recurrence risk using the six machine learning models are presented in Table 3. DeLong’s test was used to compare the predictive efficacy among the six machine learning models. There were no statistically significant differences in predictive efficacy between the random forest and logistic regression models (P value was 0.43), suggesting that their predictive capabilities were comparable and that the random forest model was superior to the remaining four models (all P values were < 0.05). To better present the results, the Delong test results were visualized using a heatmap, as shown in Fig. 3.

**Table 3:** Metrics for predicting 5-year recurrence risk in training and testing cohorts using six machine learning models

**model_name**	**Cohort**	**Accuracy**	**AUC**	**95%CI**	**Sensitivity**	**Specificity**	**PPV**	**NPV**
logistic regression	train	0.790	0.830	0.7747–0.8781	0.615	0.887	0.753	0.805
	test	0.696	0.823	0.7650 −0.8710	0.794	0.597	0.667	0.740
random forest	train	0.941	0.995	0.9911–0.9990	0.846	0.995	0.989	0.920
	test	0.776	0.845	0.7902–0.8980	0.730	0.823	0.807	0.750
XGBoost	train	0.800	0.821	0.7712–0.8740	0.712	0.849	0.725	0.840
	test	0.648	0.651	0.5932–0.7094	0.698	0.597	0.638	0.661
SVM	train	0.952	0.990	0.9606–0.9982	0.875	0.995	0.989	0.934
	test	0.712	0.752	0.6918–0.8069	0.825	0.597	0.675	0.771
BP NET	train	0.879	0.862	0.8105–0.9092	0.740	0.957	0.906	0.868
	test	0.616	0.713	0.6554–0.7716	0.714	0.516	0.600	0.640
KNN	train	0.900	0.969	0.9310–0.9878	0.760	0.978	0.952	0.879
	test	0.688	0.756	0.7018–0.8114	0.603	0.774	0.731	0.658

### Visualization of the optimal machine learning model using SHAP

**Fig. 4:**
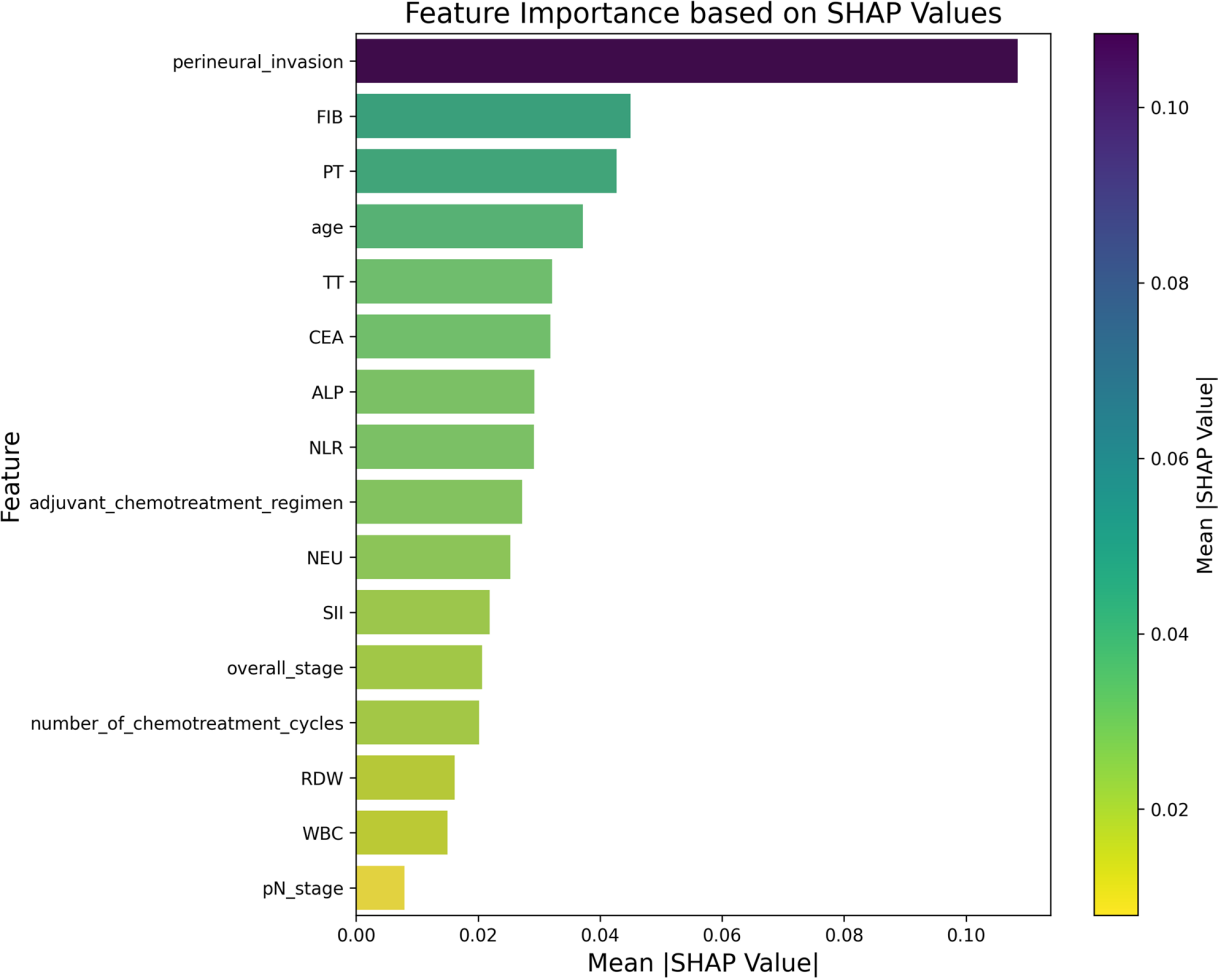
Bar chart of feature importance ranking for the random forest model

**Fig. 5:**
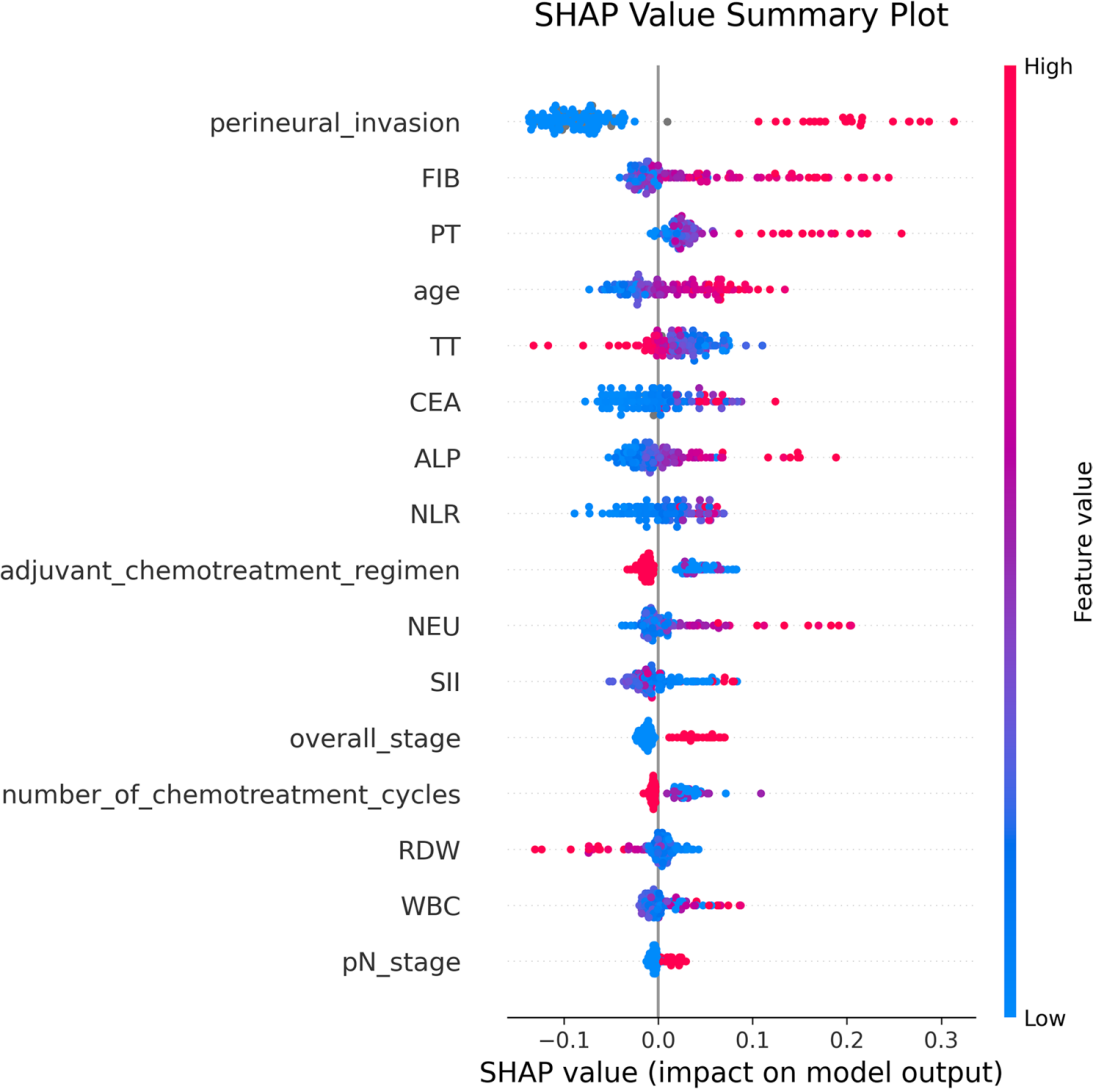
SHAP values of each sample under different feature values

The optimal random forest model was selected for analysis. A feature importance plot was constructed for the random forest model to analyse the importance of each feature in the machine learning model. The details and code for calculating SHAP values are provided in supplementary materials B. The feature importance plot indicated that the top five features were perineural invasion, FIB, PT, age, and TT, in sequential order, as depicted in Fig. 4. In the SHAP plot, each point corresponds to a patient, with feature values indicated by colour. The colour gradient from red to blue signifies feature values ranging from high to low. The SHAP value on the horizontal axis represents the predicted probability of a patient’s 5-year recurrence. A higher probability correlates with a larger SHAP value, as illustrated in Fig. 5. Features such as perineural invasion, FIB, PT, age, CEA, pN stage, and overall stage were positively correlated with recurrence, whereas features such as TT, RDW, adjuvant chemotherapy regimen, and the number of chemotherapy cycles were negatively correlated.

## Discussion

CC, a prevalent malignant tumour, has emerged as a major challenge in the realm of global public health, given its high incidence and mortality rates [21]. This underscores the need for strengthened preventive measures, strategies for early detection, and advancements in treatment methods to mitigate its significant impact on public health. Stage III CC typically involves deep infiltration of the colonic wall and adjacent lymph nodes but without distant metastasis [22]. At this stage, treatment usually requires a comprehensive approach that combines radical surgery and adjuvant chemotherapy [7, 23]. Nevertheless, the prognosis for stage III CC remains unsatisfactory, with a high recurrence rate [24]. Accurately predicting patients’ treatment outcomes has become a major challenge. Therefore, designing precise prediction tools to determine patients’ posttreatment recurrence risk is crucial for improving patient outcomes.

Several studies have been conducted on the prediction of postoperative recurrence in patients with stage III CC in China and other countries. As early as 2015, the CRC Subtyping Consortium reported a novel classification system for CC, which aids in more accurately understanding tumour biological behaviour, guiding treatment selection, and predicting prognosis [25]. Unfortunately, this molecular classification system relies on molecular characteristics (such as gene expression, mutation profiles, and epigenetic features), which restricts its universal clinical application. Mitsunori Ushigome et al. enrolled 233 stage III CC patients and identified the CRP, CEA, CA199, and T4 stages as independent prognostic factors for relapse-free survival (RFS) through multivariate analysis [26]. Matsuoka H et al. conducted a retrospective analysis of 120 stage III CRC patients who underwent curative colectomy and identified preoperative bowel obstruction, N2, and fewer than 17 examined lymph nodes as high-risk factors for recurrence [27]. Moreover, indicators calculated through laboratory tests, such as the PNI and NLR, serve as independent predictors of recurrence risk in patients with CRC [28, 29]. However, the above studies predominantly considered clinical and pathological features, either not incorporating laboratory tests or including only a small number of them.

This study integrated 48 laboratory test indicators and 15 clinical and pathological features, including postoperative adjuvant therapy information, to comprehensively evaluate preoperative patients’ multidimensional parameters and predict the recurrence risk of stage III CC. To exclude features with low correlation, univariate analysis was performed first, ultimately screening out 10 laboratory tests and 6 clinical and pathological features (as shown in the Results Section). Six different machine learning methods were employed to construct predictive models, and their performance was evaluated on the test dataset. Additionally, the DeLong test was used to analyse differences between models. Owing to the differences in the logic and complexity of various machine learning algorithms, there may also be variations in their clinical applications. In this study, the random forest model demonstrated the best predictive performance, outperforming the LR, SVM, KNN, BP NET, and XGBoost models. Its AUC reached 0.845, with a prediction accuracy of 77.6%. Random forest is an ensemble learning method that involves constructing multiple decision trees or regression trees and making predictions by averaging the predictions from all the individual trees. It leverages the power of aggregating results from various trees to improve the overall predictive performance and reduce overfitting, making it a robust and accurate model for classification and regression tasks. Previous studies have also shown that the random forest model has excellent performance in clinical applications [30, 31]. The suboptimal performance of the XGBoost model may be attributed to its poor adaptability to the current dataset; its performance is expected to improve with larger sample sizes. In contrast, logistic regression is more suitable for primary hospitals due to its advantages of easy operation, fast computation, and interpretable results. The nomogram based on logistic regression is provided in the supplementary materials C. By comparison, the random forest model requires programming tools and professional deployment, making it more appropriate for secondary and tertiary hospitals.

The SHAP algorithm, a game theory-based method, elucidates features and models by calculating the contribution of each feature to the prediction outcomes. In past studies, the SHAP method has been utilized to gain a clear understanding of the decision-making process of machine learning models. For example, Wang Y et al. applied the SHAP method to interpret a multiparameter magnetic resonance imaging radiomic model predicting the efficacy of neoadjuvant chemotherapy in patients with advanced rectal cancer [32]. In this study, the SHAP algorithm was used to provide a visual explanation of the random forest prediction model. Feature importance analysis showed that perineural invasion significantly contributed to the risk of recurrence in patients with stage III colon cancer, which aligns with the findings of previous studies [33]. The adjuvant chemotherapy regimen and the number of chemotherapy cycles were negatively correlated with 5-year recurrence. In other words, combined chemotherapy and a full course of chemotherapy can reduce the risk of recurrence.

In recent years, deep learning techniques have also been applied to the field of colorectal cancer recurrence prediction. Domestic researchers have identified high-risk patients with locally advanced colorectal cancer based on CT images [34], achieving an AUC of approximately 0.85, which is comparable to the performance observed in our study. International scholars have combined pathological images with ctDNA testing to predict postoperative recurrence risk in colorectal cancer, yielding an AUC of 0.9 [35]. However, the widespread application of ctDNA is limited by its high detection costs. Moving forward, integrating imaging and pathological images into our current research framework could enable more accurate prediction of recurrence risk. Additionally, one aspect warrants further attention throughout the process. Several research reports have been published regarding other tumor types [36, 37]. The AUC of the random forest model on the training set was nearly 1, which raises concerns about potential overfitting. Subsequently, the 5-fold cross-validation we conducted revealed that the error rate fluctuated between 0.25 and 0.30, and the accuracy fluctuated between 0.70 and 0.75. For the independent test set, the AUC reached 0.845. Therefore, we assessed the model’s performance as good, with strong generalization ability. A similar situation was also reported in a study by Barrenada L [38]. This study included two cohorts with an acceptable sample size; however, it lacked validation data from multiple centres with larger sample sizes. Consequently, further optimization using multicentre datasets with larger sample sizes is essential to enhance accuracy and generalizability.

## Conclusion

Among the six machine learning models constructed using preoperative laboratory tests and clinical and pathological features, the random forest model demonstrated optimal performance and the best model stability. This model can assist in predicting the 5-year recurrence risk of patients with stage III CC, providing potential reference value for the clinical development of individualized treatment strategies. Moreover, the machine learning model can be interpreted through the SHAP method, which helps in understanding the decision-making process of the model.

## Supplementary Information

Supplementary Material 1. Table S1: Results of Multiple Imputation for Features with Missing Data. Table S2. Univariate Analysis of Clinical and Pathological Features and Laboratory Tests in the Training Set (P-values ≥ 0.05). Table S3. The result of the random forest with five-fold cross-validation.

Supplementary Material 2. Figure S1. Logistic Regression-Based Nomogram Prediction Model for 5-Year Postoperative Recurrence Risk in Patients with Stage III Colon Cancer.

## Data Availability

The dataset was collected from the hospital’s case management system, and all data are in the authors’ possession.

## Abbreviations

CRC: Colorectal cancer
CC: Colon cancer
SVM: Support vector machine
BP NET: Back propagation neural network
KNN: K-nearest neighbour
AUC: Area under the curve
SHAP: Shapley additive explanations
BMI: Body mass index
PNI: Prognostic nutritional index
SII: Systemic immunoinflammatory index
PLR: Platelet-to-lymphocyte ratio
NLR: Neutrophil-to-lymphocyte ratio
RFS: Relapse-free survival

## References

[1] Morgan E, Arnold M, Gini A, et al. Global burden of colorectal cancer in 2020 and 2040: incidence and mortality estimates from GLOBOCAN. Gut. 2023;72(2):338–44. 36604116 10.1136/gutjnl-2022-327736

[2] Patel SG, Karlitz JJ, Yen T, et al. The rising tide of early-onset colorectal cancer: a comprehensive review of epidemiology, clinical features, biology, risk factors, prevention, and early detection. Lancet Gastroenterol Hepatol. 2022;7(3):262–74. 35090605 10.1016/S2468-1253(21)00426-X

[3] Oggesen BT, Hamberg MLS, Rosenberg J. Practical management algorithms for late complications after colorectal and anal cancer—Basic treatment of late complications. Med Adv. 2023;1(3):260–9.

[4] Fadlallah H, El Masri J, Fakhereddine H, et al. Colorectal cancer: recent advances in management and treatment. World J Clin Oncol. 2024;15(9):1136–56. 39351451 10.5306/wjco.v15.i9.1136 PMC11438855

[5] Fabregas JC, Ramnaraign B, George TJ. Clinical updates for colon cancer care in 2022. Clin Colorectal Cancer. 2022;21(3):198–203. 35729033 10.1016/j.clcc.2022.05.006

[6] Dhimal T, Fleming FJ. Neoadjuvant immunotherapy in operable colon cancer: opportunities and challenges. Ann Surg Oncol. 2025;32(5):3049–51. 39988723 10.1245/s10434-025-17043-z

[7] Benson AB, Venook AP, Adam M et al. Colon Cancer, version 3.2024, NCCN clinical practice guidelines in oncology. J Natl Compr Canc Netw. 2024;22(2 D). 10.6004/jnccn.2024.0029 38862008

[8] Gately L, Jalali A, Semira C, et al. Stage dependent recurrence patterns and post-recurrence outcomes in non-metastatic colon cancer. Acta Oncol. 2021;60(9):1106–13. 34184594 10.1080/0284186X.2021.1943519

[9] Ryu HS, Kim J, Park YR et al. Recurrence patterns and risk factors after curative resection for colorectal cancer: insights for postoperative surveillance strategies. Cancers (Basel). 2023;15(24). 10.3390/cancers15245791 PMC10742009 38136337

[10] Henriksen TV, Tarazona N, Frydendahl A, et al. Circulating tumor DNA in stage III colorectal Cancer, beyond minimal residual disease Detection, toward assessment of adjuvant therapy efficacy and clinical behavior of recurrences. Clin Cancer Res. 2022;28(3):507–17. 34625408 10.1158/1078-0432.CCR-21-2404 PMC9401484

[11] Jiang W, Wang H, Dong X, et al. Pathomics signature for prognosis and chemotherapy benefits in stage III colon cancer. JAMA Surg. 2024;159(5):519–28. 38416471 10.1001/jamasurg.2023.8015 PMC10902777

[12] Nors J, Iversen LH, Erichsen R, et al. Incidence of recurrence and time to recurrence in stage I to III colorectal cancer: A nationwide Danish cohort study. JAMA Oncol. 2024;10(1):54–62. 37971197 10.1001/jamaoncol.2023.5098 PMC10654928

[13] Ye H, Ye Y, Wang Y, et al. Automated assessment of necrosis tumor ratio in colorectal cancer using an artificial intelligence-based digital pathology analysis. Med Adv. 2023;1(1):30–43.

[14] Xu C, Xia P, Li J, et al. Discovery and validation of a 10-gene predictive signature for response to adjuvant chemotherapy in stage II and III colon cancer. Cell Rep Med. 2024;5(8):101661. 39059386 10.1016/j.xcrm.2024.101661 PMC11384724

[15] Muhammad D, Ahmed I, Ahmad MO et al. Randomized explainable machine learning models for efficient medical diagnosis. IEEE J Biomed Health Inf. 2024;PP. 10.1109/JBHI.2024.3491593 40030196

[16] Greener JG, Kandathil SM, Moffat L, et al. A guide to machine learning for biologists. Nat Rev Mol Cell Biol. 2022;23(1):40–55. 34518686 10.1038/s41580-021-00407-0

[17] Yan J, Liu L, Wang W, et al. Radiomic features from Multi-Parameter MRI combined with clinical parameters predict molecular subgroups in patients with Medulloblastoma. Front Oncol. 2020;10:558162. 33117690 10.3389/fonc.2020.558162 PMC7566191

[18] Liu Z, Hong X, Wang L, et al. Radiomic features from multiparametric magnetic resonance imaging predict molecular subgroups of pediatric low-grade gliomas. BMC Cancer. 2023;23(1):848. 37697238 10.1186/s12885-023-11338-8 PMC10496393

[19] Erciyestepe M, Selvi O, Dinc G, et al. Factors affecting recurrence and survival in stage IIA colon cancer patients. Oncology. 2024;102(12):1009–17. 39008971 10.1159/000540334

[20] Maruyama T, Shimoda M, Hakoda H, et al. Preoperative prognostic nutritional index predicts risk of recurrence after curative resection for stage IIA colon cancer. Am J Surg. 2021;222(1):179–85. 33138968 10.1016/j.amjsurg.2020.10.032

[21] Horesh N, Emile SH, Garoufalia Z, et al. Trends in management and outcomes of colon cancer in the united States over 15 years: analysis of the National cancer database. Int J Cancer. 2024;155(1):139–48. 38454540 10.1002/ijc.34910

[22] Zeng H, Zhong X, Liu W, et al. Predicting treatment failure in stage III colon cancer patients after radical surgery. Front Oncol. 2024;14:1397468. 38817900 10.3389/fonc.2024.1397468 PMC11137277

[23] Lee KH, Park SY, Song SH et al. Short-term outcomes of early versus conventional adjuvant chemotherapy in stage III colon cancer: randomized clinical trial. BJS Open. 2023;7(4). 10.1093/bjsopen/zrad064 PMC10338899 37439066

[24] Liu B, Zhang ZX, Nie XY, et al. Clinical outcome and prognostic factors of T4N0M0 colon cancer after R0 resection: A retrospective study. World J Gastrointest Oncol. 2024;16(5):1869–77. 38764842 10.4251/wjgo.v16.i5.1869 PMC11099430

[25] Hu F, Wang J, Zhang M, et al. Comprehensive analysis of Subtype-Specific molecular characteristics of colon cancer: specific Genes, driver Genes, signaling Pathways, and immunotherapy responses. Front Cell Dev Biol. 2021;9:758776. 34912802 10.3389/fcell.2021.758776 PMC8667669

[26] Nakamura S, Kageyama H, Torii K, et al. High-risk features for recurrence in patients with stage III colorectal cancer: A retrospective cohort study. Anticancer Res. 2024;44(2):757–66. 38307588 10.21873/anticanres.16867

[27] Matsuoka H, Ando K, Hu Q, et al. Postoperative C-reactive protein/albumin ratio is a biomarker of risk of recurrence and need for adjuvant chemotherapy for stage III colorectal cancer. Int J Clin Oncol. 2020;25(7):1318–26. 32279124 10.1007/s10147-020-01672-3

[28] Shida D, Inoue M, Tanabe T, et al. Prognostic impact of primary tumor location in stage III colorectal cancer-right-sided colon versus left-sided colon versus rectum: a nationwide multicenter retrospective study. J Gastroenterol. 2020;55(10):958–68. 32651860 10.1007/s00535-020-01706-7

[29] Qiu J, Yu Y, Wang Z, et al. Developing individualized Follow-Up strategies based on High-Risk recurrence factors and dynamic risk assessment for locally advanced rectal cancer. Cancer Med. 2024;13(20):e70323. 39467147 10.1002/cam4.70323 PMC11516045

[30] Yan L, Ye Q, Shi B, et al. Random forest-based model for the recurrence prediction of borderline ovarian tumor: clinical development and validation. J Cancer Res Clin Oncol. 2025;151(5):160. 40349260 10.1007/s00432-025-06221-x PMC12066375

[31] Mendapara K. Development and evaluation of a chronic kidney disease risk prediction model using random forest. Front Genet. 2024;15:1409755. 38993480 10.3389/fgene.2024.1409755 PMC11236722

[32] Wang Y, Zhang L, Jiang Y, et al. Multiparametric magnetic resonance imaging (MRI)-based radiomics model explained by the Shapley additive explanations (SHAP) method for predicting complete response to neoadjuvant chemoradiotherapy in locally advanced rectal cancer: a multicenter retrospective study. Quant Imaging Med Surg. 2024;14(7):4617–34. 39022292 10.21037/qims-24-7 PMC11250347

[33] Guo T, Cheng B, Li Y, et al. A radiomics model for predicting perineural invasion in stage II-III colon cancer based on computer tomography. BMC Cancer. 2024;24(1):1226. 39367321 10.1186/s12885-024-12951-x PMC11453003

[34] Zhou Y, Zhao J, Tan Y, et al. CT-based radiomics deep learning signatures for noninvasive prediction of early recurrence after radical surgery in locally advanced colorectal cancer: A multicenter study. Eur J Surg Oncol. 2025;51(12):110482. 41014758 10.1016/j.ejso.2025.110482

[35] Loeffler CML, Bando H, Sainath S, et al. HIBRID: histology-based risk-stratification with deep learning and ctdna in colorectal cancer. Nat Commun. 2025;16(1):7561. 40813777 10.1038/s41467-025-62910-8 PMC12354865

[36] Sun Q, Chen Y, Liang C, et al. Biologic pathways underlying prognostic radiomics phenotypes from paired MRI and RNA sequencing in glioblastoma. Radiology. 2021;301(3):654–63. 34519578 10.1148/radiol.2021203281

[37] Zhao Y, Liu G, Sun Q, et al. Validation of CT radiomics for prediction of distant metastasis after surgical resection in patients with clear cell renal cell carcinoma: exploring the underlying signaling pathways. Eur Radiol. 2021;31(7):5032–40. 33439312 10.1007/s00330-020-07590-2

[38] Barrenada L, Dhiman P, Timmerman D, et al. Understanding overfitting in random forest for probability estimation: a visualization and simulation study. Diagn Progn Res. 2024;8(1):14. 39334348 10.1186/s41512-024-00177-1 PMC11437774

