# Supplementary material for: Interpretable machine learning model for predicting 5-Year postoperative recurrence risk in patients with stage III colon cancer using preoperative laboratory tests: a two-centre study
Source: BMC Gastroenterol. 2026 Jan 29;26:72. doi: 10.1186/s12876-025-04511-9 (PMC12857113; doi:10.1186/s12876-025-04511-9)
Supplement: Supplementary file 2 — Supplementary Material 2. Figure S1. Logistic Regression-Based Nomogram Prediction Model for 5-Year Postoperative Recurrence Risk in Patients with Stage III Colon Cancer. [file 12876_2025_4511_MOESM2_ESM.doc]

**Supplementary Materials A**

Six Specific parameters of the six machine learning models:

Logistic regression: No parameter tuning

Support Vector Machine (SVM):$cost[1] 3

$gamma[1] 0.1

Random Forest:$mtry[1] 2

$ntree[1] 350

K-Nearest Neighbors (KNN):$k[1] 5

$kernel[1] "rectangular"

$distance[1] 2.111111

Backpropagation Neural Network (BP Neural Network):$size[1] 2

$decay[1] 0.1

$maxit[1] 300

Extreme Gradient Boosting (Xgboost):$eta[1] 0.05

$max_depth[1] 4

$gamma[1] 3

**Supplementary Materials B**

Functions and code for SHAP calculation: Implemented using the shap and sklearn packages in Python.

import pandas as pd

import numpy as np

import matplotlib.pyplot as plt

import seaborn as sns

from sklearn.ensemble import RandomForestRegressor

import shap

import os

# Set file paths

train_path = "D:/XL0522_290_new.xlsx"

test_path = "D:/YZ0522_125.xlsx"

shap_output_path = "D:/shap_values.xlsx"

summary_plot_path = "D:/shap_summary_plot.png"

bar_plot_path = "D:/shap_bar_plot.png"

# Load data

train_data = pd.read_excel(train_path)

test_data = pd.read_excel(test_path)

# Remove ID column

train_data = train_data.drop('ID', axis=1, errors='ignore')

test_data = test_data.drop('ID', axis=1, errors='ignore')

# Separate features and target variable (RFS as the target)

X_train = train_data.drop('RFS', axis=1)

y_train = train_data['RFS']

X_test = test_data.drop('RFS', axis=1)

y_test = test_data['RFS']

# Train the random forest model

model = RandomForestRegressor(n_estimators=200,

max_depth=10,

min_samples_split=5,

random_state=42,

n_jobs=-1)

model.fit(X_train, y_train)

# Calculate predicted values for the test set

y_pred = model.predict(X_test)

# Compute SHAP values

explainer = shap.TreeExplainer(model)

shap_values = explainer(X_test)

# Save SHAP values to Excel

shap_df = pd.DataFrame(shap_values.values, columns=X_test.columns)

shap_df.to_excel(shap_output_path, index=False)

Table S1: Results of Multiple Imputation for Features with Missing Data.

Table S2. Univariate Analysis of Clinical and Pathological Features and Laboratory Tests in the Training Set (P-values ≥ 0.05).

Table S3. The result of the random forest with five-fold cross-validation.

**Supplementary Materials C**

Figure S1. Logistic Regression-Based Nomogram Prediction Model for 5-Year Postoperative Recurrence Risk in Patients with Stage III Colon Cancer.
